# The Random Soldier Kinematogram: a novel tool for task-switching research in military context

**DOI:** 10.3389/fpsyg.2026.1678090

**Published:** 2026-03-13

**Authors:** Sami Mecheri, Wendie Gouasmi, Baptiste Dozias, Régis Lobjois

**Affiliations:** 1 Département Neurosciences et Sciences Cognitives, Institut de Recherche Biomédicale des Armées, Brétigny-sur-Orge, France; 2 COSYS-PICS-L, Université Gustave Eiffel, Marne-la-Vallée, France

**Keywords:** attention, cognitive control, military, multitasking, task switching

## Abstract

Adaptive behavior in high-stakes, multitasking environments requires flexible switching between tasks with changing circumstances. However, task switching has typically been studied using static, simple, and unnoisy stimuli that are unrepresentative of most real-world operational environments. Here, we developed the Random Soldier Kinematogram (RSK), an array of moving soldiers, to better reflect the processing demands of military operational environments with dynamic, complex, and noisy stimuli. Across three experiments, we introduced RSK motion (discriminating the coherent motion direction: leftward/rightward) and color (discriminating the majority uniform color: khaki/white) tasks, by examining their cognitive-control demands in single-task blocks (Experiments 1 and 2) and a task-switching context (Experiment 3). Experiment 1 ruled out the possibility that perceptual differences between uniform colors involved different discrimination performances. Experiment 2 orthogonally varied motion coherence and color coherence to gain insight into participants' sensitivity to target (task-relevant) and distractor (task-irrelevant) information. Results showed unequal task strength, with a motion task that is less control-demanding (dominant) and a color task that is more control-demanding (non-dominant), to overcome interference from motion. Experiment 3 investigated the efficiency of cognitive control processes involved in switching between RSK tasks (switch costs) and maintaining competing task-sets (mixing costs). Asymmetric switch cost was found, with larger cost when switching to the motion (dominant) task, since stronger cognitive effort was necessary to release it from its backward inhibition. We conclude that RSK expands possibilities for task-switching research in the military by better sampling soldiers' surrounding informational constraints with adjustable signal-to-noise ratios of task-relevant and task-irrelevant information.

## Introduction

Goal-directed behavior in complex environments requires the flexible reconfiguration of task-sets (the stimulus-response rules that define a task, Monsell, 2003) in response to changing goals or circumstances. A widely used approach to study such flexibility is the task-switching paradigm, in which participants either repeat the same task or switch between different tasks (for a review, see Grange and Houghton, 2014). A critical and robust finding in task-switching research is that the execution of sequential tasks incurs a performance cost when switching from one task to another, manifested in increased response times and error rates (Wylie and Allport, 2000). This switch cost persists after practice (Stoet and Snyder, 2007), time to prepare for the upcoming switch (Allport et al., 1994), and knowledge of the upcoming task (Sohn and Carlson, 2000), indicating that switching requires time-consuming cognitive control to reconfigure the newly relevant task-set (Meiran, 1996; Rogers and Monsell, 1995) and/or to resolve interference from the previously active task-set (Kiesel et al., 2010; Vandierendonck et al., 2010). While the costs of switching constrain performance in multitasking and complex environments—such as military settings—research on task switching has relied primarily on stimuli that do not reflect the processing demands of most operational contexts (Chérif et al., 2018). In the present study, we introduce novel tasks designed to better reflect the visual processing demands of situations in which soldiers make high-stakes, time-sensitive decisions across sequential tasks.

The primary goal of the task-switching literature has been to elucidate the cognitive architecture of switch costs (Koch et al., 2018), and thus the studies have generally involved static, simple and unnoisy stimuli that are highly controlled but unrepresentative of military environments. This disparity is particularly evident for dismounted soldiers operating on foot in the field (as opposed to in armored vehicles, helicopters, or other modes of transport), as they directly engage the enemy, secure areas, conduct reconnaissance, and gather intelligence on the ground. In such contexts, soldiers frequently encounter unexpected stimuli due to incomplete battlefield information, making the ability to flexibly reconfigure task-sets crucial. One key disparity is that conventional studies typically require participants to process simple stimuli (shapes, letters, or digits) rather than complex visual scenes (Draheim et al., 2016). This contrasts with dismounted situations, where soldiers must filter a profusion of both task-relevant and task-irrelevant information. A second difference is the use of static stimuli in conventional studies, whereas dismounted environments involve dynamic stimuli that unfold unpredictably. A third key difference is the conventional reliance on stimuli with unambiguous correct responses (which of two categories a stimulus belongs to, such as square/oval for shapes, vowel/consonant for letters, or odd/even for digits). In contrast, soldiers often make decisions based on noisy target information (obstructed views, camouflage, low illumination; see Cockrell, 1979). These disparities make it challenging to generalize findings from conventional task-switching studies to military-relevant tasks. For instance, recent research has demonstrated that another robust task-switching finding—the N-2 repetition cost, which reflects persisting inhibition of abandoned tasks—is no longer observed when more naturalistic and complex stimulus material is used (Schuch et al., 2020). This highlights the importance of developing tasks that better reflect the visual processing demands of operational environments, incorporating dynamic, complex and noisy stimuli, for studying task-switching performance in the military.

To achieve this goal, we developed the Random Soldier Kinematogram (RSK), which extends the Random Dot Kinematogram (RDK, Danielmeier et al., 2011; Shenhav et al., 2018) in order to present soldiers as stimuli objects instead of dots. The RDK has been used for decades to study motion perception (Braddick, 1980; Julesz, 1971) and perceptual decision-making (Kayser et al., 2010; Mante et al., 2013; Shadlen and Newsome, 1996). In RDK paradigms, some dots move coherently in a specific direction, while other dots move randomly, and participants are asked to indicate the direction of coherent motion (e.g., up or down). A key feature of the RDK is its flexibility in varying coherence levels from 0% (all dots move randomly) to 100% coherence (all dots move uniformly in one direction), enabling the construction of psychometric curves relating motion coherence to performance. Prior studies have reported a non-linear increase in accuracy with increasing coherence, saturating above chance performance (Baker et al., 1991; Lankheet and Verstraten, 1995). Similarly, reaction times decrease as coherence increases (Strittmatter et al., 2023; Spitzer et al., 2022, 2019). The RSK consists of a crowd of soldiers wearing khaki or white uniforms, with some soldiers moving coherently in one horizontal direction (leftward or rightward) and others moving randomly. This allows participants to respond to two different tasks: categorizing the color of the RSK regardless of its motion (task goal: discriminating the majority uniform color), and categorizing the motion of the RSK regardless of its color (task goal: discriminating the direction of coherent motion). To capture noisy elements in stimuli processing, RSK parametrically varies motion coherence (i.e., percentage of soldiers moving leftward or rightward) and color coherence (i.e., percentage of the majority uniform color). This approach provides two methodological advantages. First, introducing noisy information enables the variation of task difficulty by changing the signal-to-noise ratio of the task-relevant stimulus dimension. Second, employing bivalent stimuli (affording responses in both tasks) and overlapping responses may allow for cross-task interference—a performance decrement when the task-irrelevant response interferes with the current response selection process (Egner, 2023)—particularly in a task-switching context. Critically, whereas stimulus-response (S-R) mapping in the color task is arbitrary, responses in the motion task are aligned with motion direction, producing spatial S-R compatibility (i.e., response keys occupy the same relative positions as the target motion directions; Kornblum et al., 1990). This may generate a difference in task strength, as the color task should not activate automatic responses and the motion task should be performed more easily.

In the present study, we introduce RSK tasks to study sequential multitasking^1^ using perceptual discrimination tasks that share some of the visual processing demands with the environments in which dismounted soldiers operate. Across three experiments, participants were required to perform RSK tasks under conditions in which they did not switch between these tasks (i.e., executing tasks in single-task blocks, Experiments 1 and 2) and in a task-switching context (Experiment 3). Experiment 1 was conducted as a preliminary step to ensure that the uniform colors, which corresponded to true military uniforms but were not perceptually equidistant, did not yield distinct processing demands. In Experiment 2, we orthogonally varied motion coherence and color coherence in each task to gain insight into participants' sensitivity to target and distractor information. This was done in the absence of task switching to prevent regularly “refreshing” distractor information by the execution of another task in which it is relevant, thus allowing for a proper assessment of cross-task interference and relative task strength. In Experiment 3, we employed a task-switching paradigm with RSK tasks at fixed coherence parameters to explore the costs of switching between these tasks and to examine how these costs relate to those typically observed in the task-switching literature.

## General method

### Participants

Forty-two participants (20 females, 22 males), 20–49 years old, volunteered to participate in this study. All reported normal or corrected-to-normal visual acuity and normal color vision. They were unaware of the hypotheses under investigation. All took part in Experiments 1 and 2 (mean age = 31 ± 8 years), and 30 of these participants took part in Experiment 3 (mean age = 34 ± 9 years). Participants were tested individually and in-person at the French Armed Forces Biomedical Research Institute. All participants were civilian and all gave informed consent before taking part in the study, which was approved by the local ethics committee.

### Stimuli

**Figure 1:**
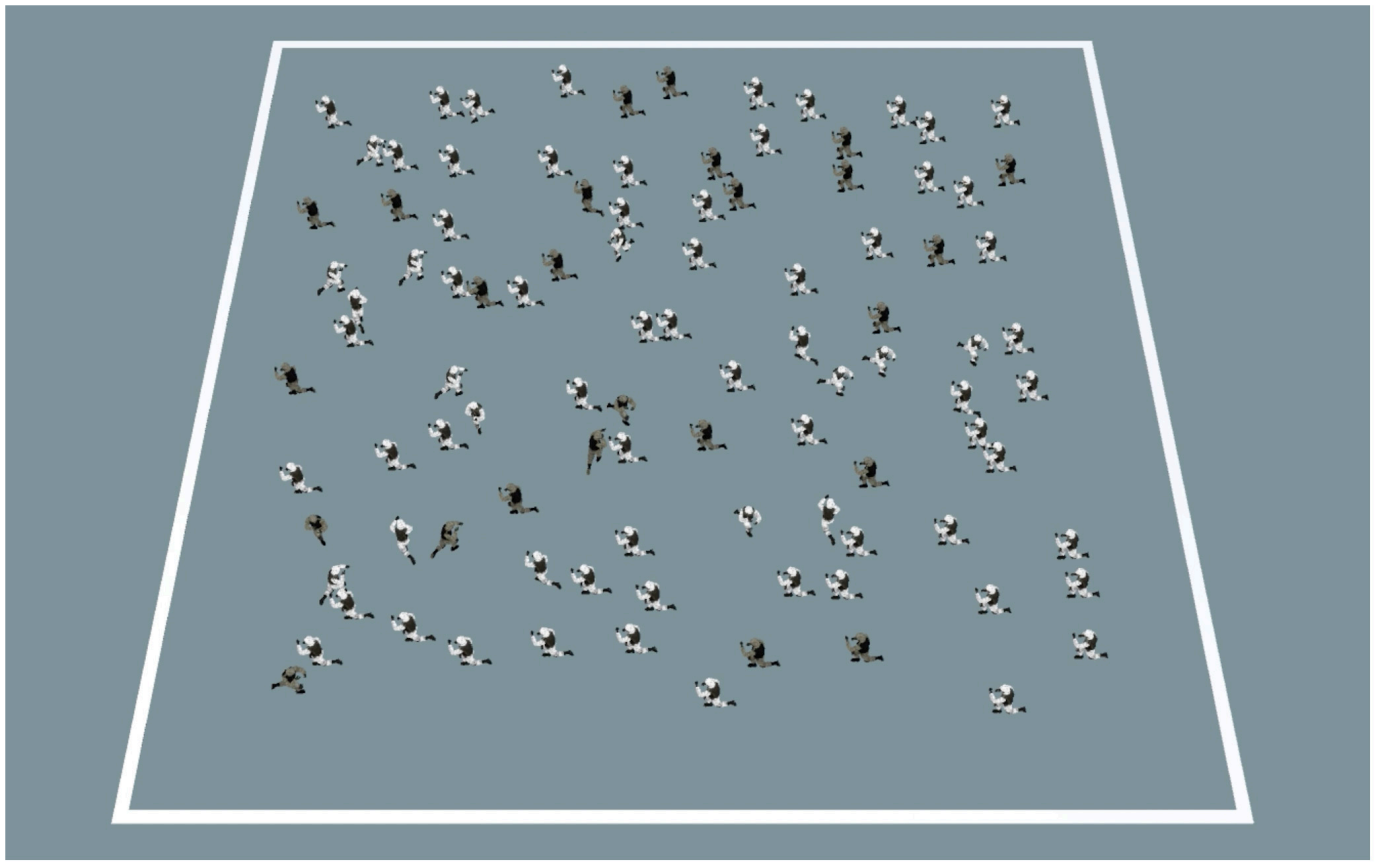
Random Soldier Kinematogram (RSK). This example frame shows an RSK with 75% motion coherence (proportion of soldiers moving horizontally; in this case, leftward) and 75% color coherence (proportion of majority uniform color; in this case, white).

Stimuli were RSKs consisting of a crowd of 100 soldiers moving on an untextured floor surface surrounded by a squared area (Figure 1). All soldiers moved on a linear path with the same running movements. The squared area was presented with an isometric camera angle from a high point, simulating an observation post. The RSK stimuli had both a motion and a color dimension. Regarding motion, a proportion of soldiers moved in the same coherent direction (horizontally leftward or rightward) and the others moved in a random direction. Regarding color, soldiers wore either khaki (“central-Europe camouflage,” typically worn in woodland areas) or white (“snow camouflage,” typically worn in mountainous regions) uniforms, with same-colored boots and vests. Both uniforms consisted of disruptive patterns with colored irregular markings.

RSKs were presented centrally on a screen (1,920 × 1,080 pixels, 75 Hz) and subtended 18° of visual angle. Speed of the moving soldiers was 5°/s. To conserve RSK density throughout a trial, soldiers were re-drawn on the opposite-side before exiting the boundaries of the squared area. Stimuli were designed using the Unity game engine and a Unity application (Unity Technologies, CA).

### RSK tasks

In the motion task, participants had to discriminate the coherent motion direction in the RSK (leftward or rightward), while ignoring the soldiers' uniform color. In the color task, participants had to discriminate the majority color uniform in the RSK (khaki or white), while ignoring the soldiers' motion path. Participants used their left and right index fingers to respond on the same response keys that were used for both tasks (“S” for left/khaki, “M” for right/white). The response keys were labeled with appropriate colors for the color task on a regular keyboard. In the color task, category-to-response mapping (“S” = khaki, “M” = white, and vice versa) was counterbalanced across participants in each experiment. Participants' responses were recorded at a frequency of 75 Hz. No feedback was provided to participants after experimental trials.

### General procedure

**Figure 2:**
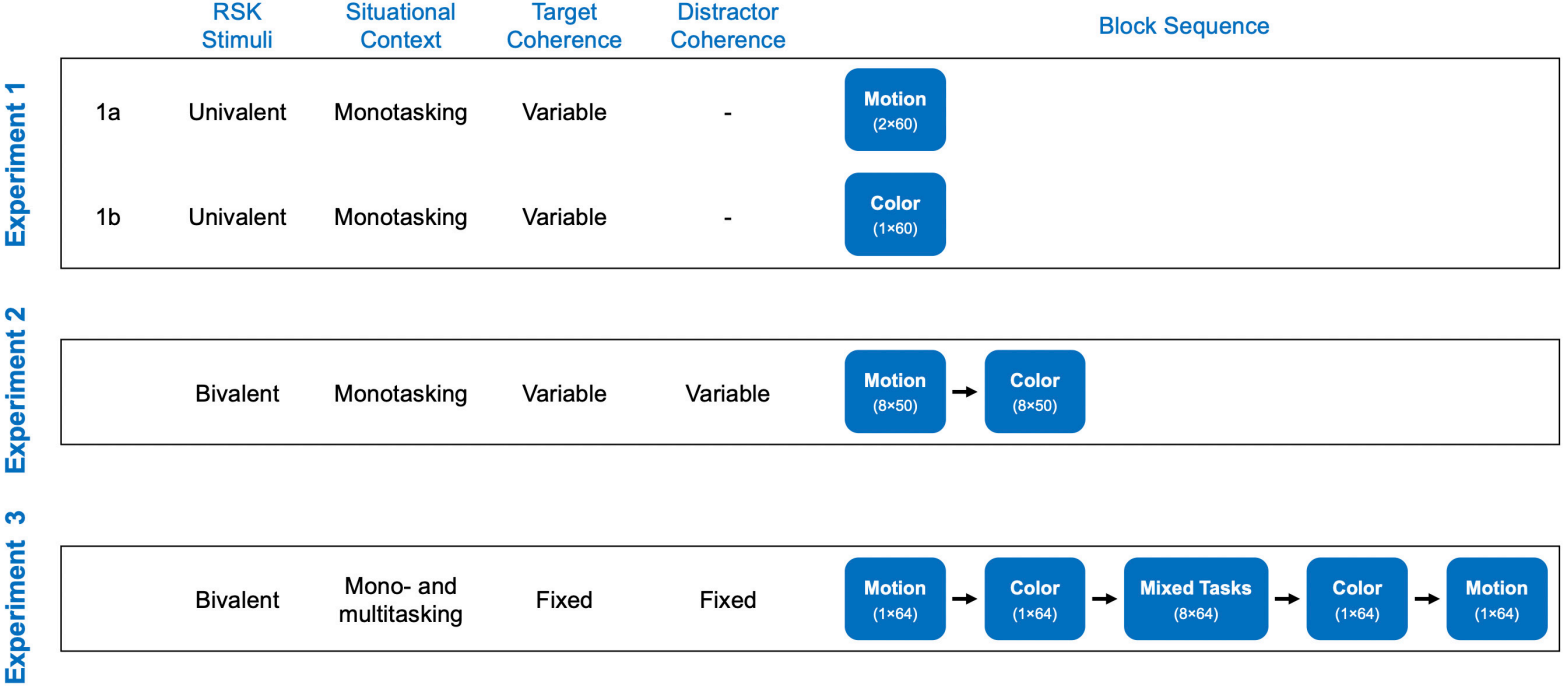
Schematic overview of the study procedure. The type of stimuli (univalent, bivalent), situational context (monotasking, multitasking), and coherence parameters (fixed, variable) are given for each experiment. The numbers given in the design of the block sequence correspond to the number of trials in each block. In Experiment 1, RSK stimuli did not involve distractor information, and the order of Experiments 1a and 1b was counterbalanced across participants. In Experiments 2 and 3, the first RSK task executed in the block sequence was counterbalanced across participants.

A schematic overview of the study procedure is depicted in Figure 2. Depending on the experiment, motion and color tasks were presented in series of single-task blocks (consisting of only one task: Experiments 1 and 2) or in series of single- and mixed-task blocks (two tasks intermixed: Experiment 3). Experiments 1 and 2 were conducted within a single experimental session (duration of around 75 min, with the order of experiments counterbalanced). Experiment 3 was carried out in another experimental session (duration of around 50 min) scheduled after a period of approximately 2 months. Video examples of stimuli are provided in Supplementary material. Participants were tested in the same room for all experiments, seated approximately 60 cm from a computer screen. At the beginning of each experiment, they received written instructions specifying the tasks to be performed and the relevant S-R mappings. They then completed practice trials before the experimental trials for task familiarization and learning of S-R mappings. After the practice trials, they were asked to verbalize the instructions to ensure full comprehension, and their performances were checked (to ensure an accuracy level of >80%). Throughout the experiments, participants were asked to respond as quickly as possible without scarifying accuracy. They could take a short pause after completing each block, and re-starting the next block was self-paced.

All data analyses were performed using JAMOVI software (version 2.5.4, JAMOVI, Sydney, NSW, Australia), implementing the “jmv” R package. Task performance was assessed through response time (RT) and accuracy. In all experiments, trials with RTs faster than 200 ms and the first trial of each block were discarded. For RT analyses, trials with incorrect responses were further excluded. For accuracy analyses, incorrect trials were counted as errors. Before all statistical analyses, RTs were log-transformed and accuracy was arcsine-transformed to improve normality and achieve greater homogeneity of variances (Hogg and Craig, 1995). ANOVA sphericity violations were explored with Mauchly's test, and a Greenhouse–Geisser correction was performed when necessary. For each significant effect, partial eta-squared ($\eta_{\text{p}}^{2}$) was calculated to determine the proportion of explained variance. *Post-hoc* analyses were conducted with Bonferroni-corrected pairwise comparisons. Descriptive statistics were reported using back-transformed means (for easier interpretation) and standard deviations. All statistical tests were done with p set at 0.05.

## Experiment 1

Experiment 1 aimed to rule out the possibility that the two uniform colors, which were not perceptually equated, played a role in task performance. Rather than altering the colors and patterns of actual military clothing, we empirically tested whether the uniform colors involved different discrimination latencies. To examine this, RSKs were made univalent (involving only one stimulus dimension) to impose minimal demands on interference control. In Experiment 1a, participants performed the motion task while color coherence was fixed at 100%. They had to discriminate the coherent motion direction in all-white or all-khaki RSKs with varying levels of motion coherence. This allowed for a comparison of motion discrimination performance between khaki- and white-uniform conditions. In Experiment 1b, participants performed the color task while motion coherence was fixed at a null value (i.e., all soldiers moved randomly). They had to discriminate the majority uniform color in RSKs with varying levels of color coherence. This allowed for a comparison of color discrimination performance between khaki- and white-majority uniforms in the absence of coherent motion. Thus, participants either executed the motion task with full color coherence (1a) or executed the color task in the absence of coherent motion (1b). It was hypothesized that the perceptual difference in colors between uniforms would not significantly affect motion or color discrimination performance.

### Method

#### Stimuli and procedure

In Experiment 1a, the color coherence was fixed at 100% (i.e., all soldiers wore khaki or white uniforms; see Table 1). For each RSK color, 10 levels of motion coherence were used (linearly spaced within 5% and 95%), each replicated six times, resulting in a total of 120 trials (motion direction was balanced across trials, with equal numbers of leftward- and rightward-moving RSKs). In Experiment 1b, the motion coherence was fixed at 0% (i.e., all soldiers moved randomly). Ten levels of color coherence were used (linearly spaced between 55% and 95%, for each majority uniform color), each replicated six times, resulting in a total of 60 trials.

**Table 1:** Parametric conditions used in Experiment 1.

**Exp**.	**Task**	**Motion coherence (%)**	**Color coherence (%)**
1a	Motion	5, 15, 25, 35, 45, 55, 65, 75, 85, 95	100_K_, 100_W_
1b	Color	0	55_K_, 65_K_, 75_K_, 85_K_, 95_K_, 55_W_, 65_W_, 75_W_, 85_W_, 95_W_

The letters in subscripts that follow color-coherence labels indicate the majority RSK uniform color (_K_ for khaki, _W_ for white).

Each trial began with a fixation cross (1,000 ms), followed by the RSK presentation (until response or 2,000 ms). The order of experiments (1a and 1b) was counterbalanced across participants. RSKs were presented in blocks of 60 trials in a pseudorandomized order with a maximum of four consecutive trials requiring the same target response button. Experimental trials were preceded by 16 practice trials.

#### Statistical analysis

In Experiment 1a, the possible effects of uniform color were assessed using 2 (RSK Color) × 10 (Motion Coherence) × 2 (Motion Direction) repeated-measures ANOVAs. A motion-direction factor was included to also control for unsuspected differences in motion discrimination between soldiers moving leftward and rightward. In Experiment 1b, the possible effects of the majority uniform color were assessed using 2 (RSK Majority Color) × 5 (Color Coherence) repeated-measures ANOVAs.

### Results

**Figure 3:**
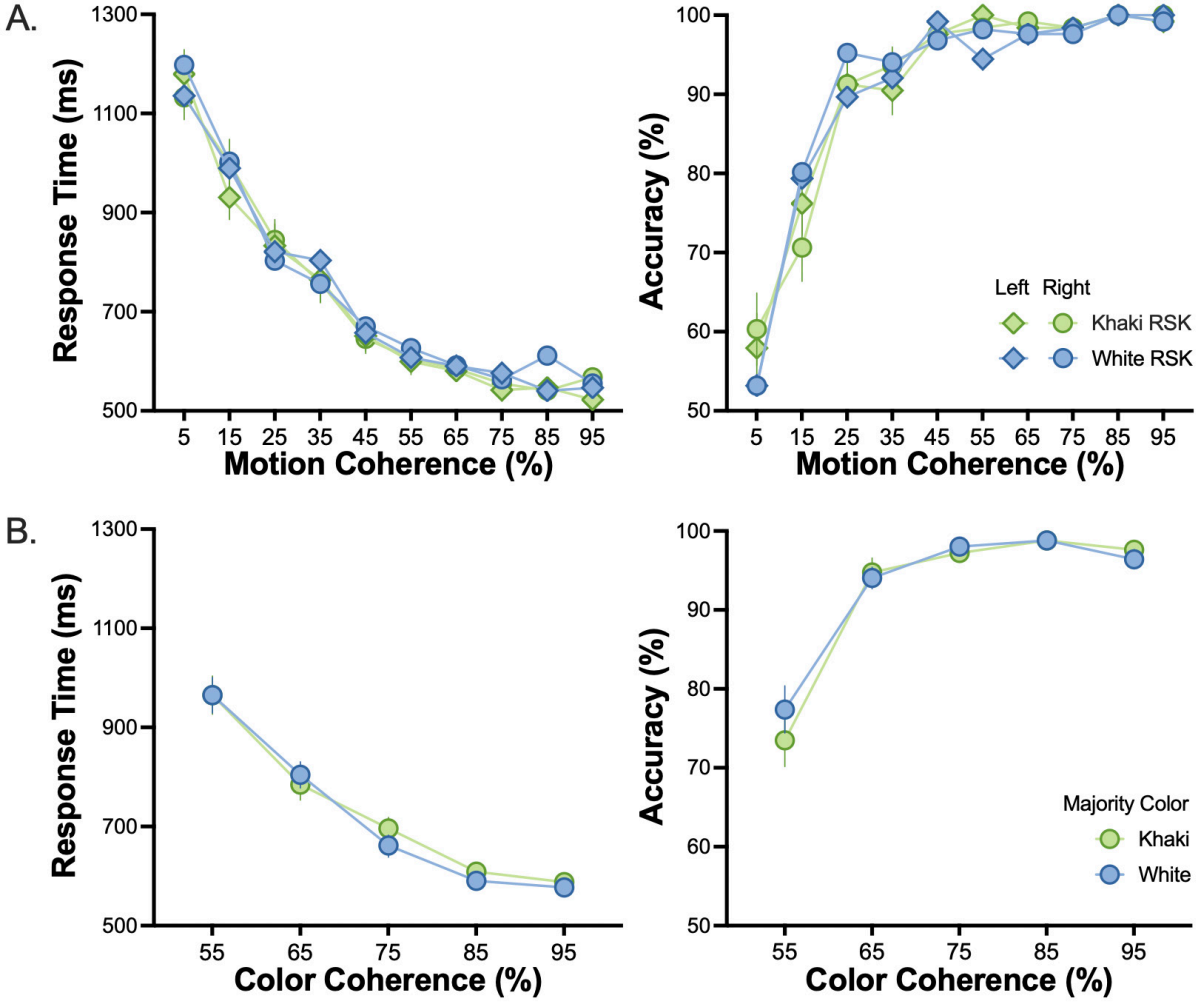
Motion and color discrimination performance for white and khaki uniform colors. **(A)** RT and accuracy in the motion task for all-white and all-khaki RSKs as a function of motion coherence and motion direction. **(B)** RT and accuracy in the color task as a function of color coherence and RSK majority uniform color. Error bars indicate the standard error of the mean.

In Experiment 1a, motion coherence had a significant main effect on RT (*F*_4.15, 157.64_ = 243.74, *p* < 0.001, $\eta_{\text{p}}^{2}$ = 0.87) and accuracy (*F*_3.77, 154.53_ = 131.11, *p* < 0.001, $\eta_{\text{p}}^{2}$ = 0.76). No other main or interaction effect was observed for these two dependent variables (see Figure 3A).

In Experiment 1b, color coherence had a significant main effect on RT (*F*_2.42, 99.36_ = 222.66, *p* < 0.001, $\eta_{\text{p}}^{2}$ = 0.84) and accuracy (*F*_3.22, 132.20_ = 67.33, *p* < 0.001, $\eta_{\text{p}}^{2}$ = 0.62). No other main or interaction effect was observed for these two dependent variables (see Figure 3B).

### Discussion

The results demonstrated no significant effect of uniform color on the discrimination of motion and color of the RSKs, in terms of both RT and accuracy. This absence of effect was true across the spectrum of coherence levels of the task-relevant dimension, for each task. In addition, soldiers' direction in the motion task had no effect on motion discrimination. Therefore, it can be concluded that khaki and white uniforms—which were not perceptually equated to preserve the characteristics of actual military clothing—involved similar discrimination response latencies and accuracies. This ruled out the possibility that perceptual differences in uniform colors would influence task performance beyond coherence parameters. It further ensured similar stimulus-driven, bottom-up processing of task-relevant and task-irrelevant information in bivalent RSKs, which were used in the subsequent experiments. Performance data (RT and accuracy) for white and khaki uniforms, as well as for leftward and rightward moving RSKs, were thus pooled for all further analyses.

## Experiment 2

Experiment 2 aimed to assess participants' sensitivity to target and distractor information of the RSK. To this end, they performed motion and color tasks with bivalent RSKs that incorporated target (task-relevant) and distractor (task-irrelevant) information. Motion coherence and color coherence were parametrically and independently manipulated in each task. This manipulation produced RSKs with congruency (motion and color signals indicating the same response) or incongruency (motion and color signals indicating opposite responses), enabling the induction of gradual degrees of task difficulty (target coherence), in addition to gradual degrees of distractor interference or facilitation. Following Ritz and Shenhav (2023), we analyzed distractor effects by parametrizing distractor congruence, between being almost fully incongruent with the correct response (e.g., in the color task: 95% rightward motion for a left color response; in the motion task: 95% left color response for a rightward-moving RSK) and being almost fully congruent (e.g., in the color task: 95% leftward motion for a left color response; in the motion task: 95% right color response for a rightward-moving RSK). By parametrizing both target coherence and distractor congruence, this experiment enhanced our understanding of task performance with bivalent RSKs and task conflict, thereby providing insight into the demands for cognitive control of each task.

Task performance was assessed by examining RT and accuracy as functions of target coherence. In line with prior works Strittmatter et al., (2023); Spitzer et al., (2022), (2019), a saturating decrease in RT was expected along with increased target coherence, as well as an increase in accuracy with increasing target coherence. Cross-task facilitation and interference were analyzed by testing the effect of distractor congruence on participants' responses. In the color task, it was expected that distracting motion would affect color discrimination. Faster and more accurate responses were thus expected when motion had higher congruence, whereas slower and less accurate responses were expected when motion had higher incongruence. In the motion task, it was expected that color would not interfere reversely with motion discrimination, due to the postulated dominance of the S-R mapping of the motion task.

### Method

#### Stimuli and procedure

**Figure 4:**
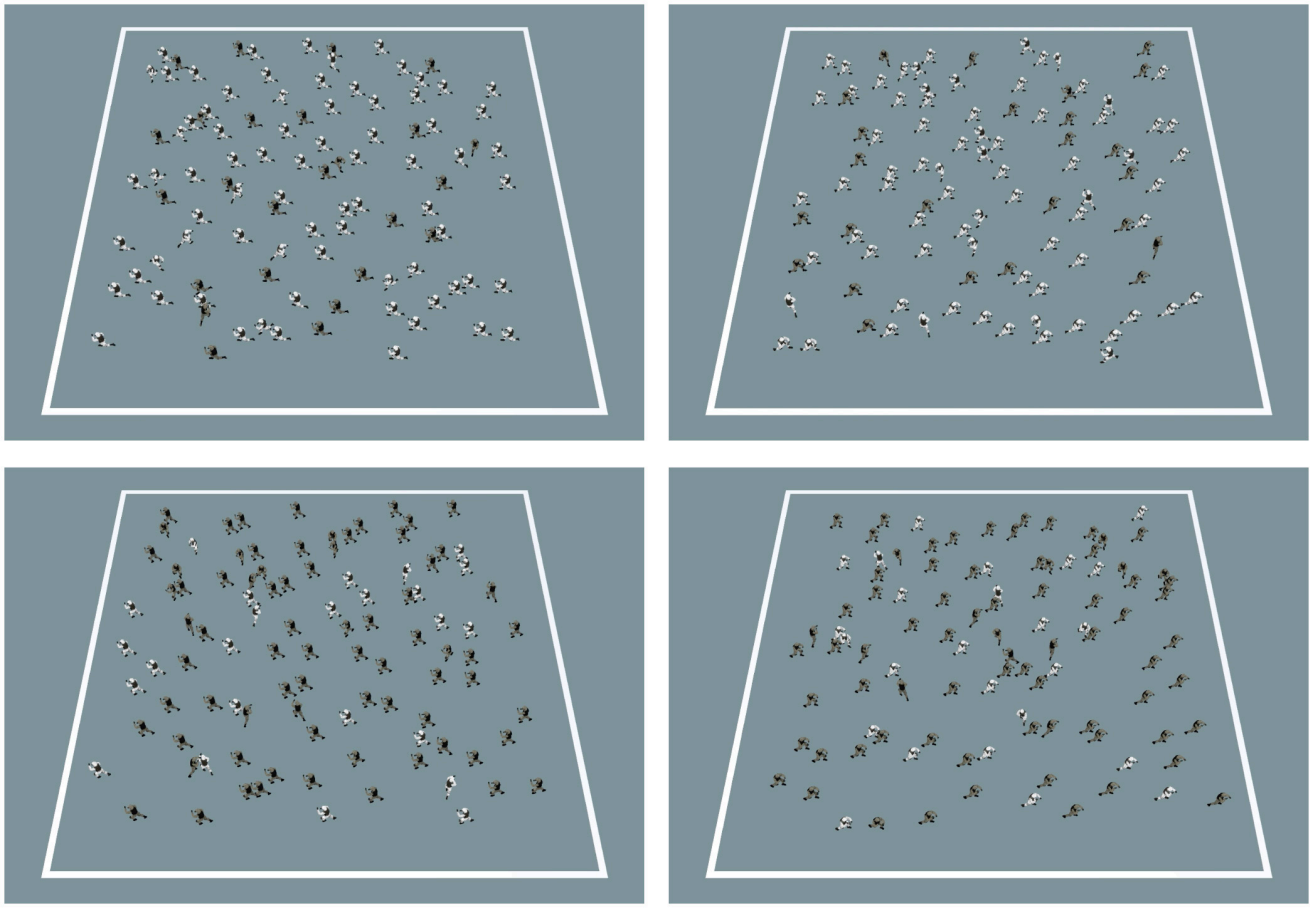
Random Soldier Kinematogram (RSK) with varying coherence parameters. Shown are RSKs with 75% motion coherence (proportion of soldiers moving horizontally) and 75% color coherence (proportion of majority uniform color). **(Left)** and **(Right)** panels represent leftward- and rightward-moving RSKs, respectively. **(Top)** panels represent RSKs with a majority of white uniforms while **(Bottom)** panels represent RSKs with a majority of khaki uniforms.

RSK stimuli consisted of combinations of 10 motion-coherence levels (linearly spaced between 5% and 95%) and 10 color-coherence levels (linearly spaced between 55 and 95%, for each majority color), making congruent and incongruent trials with equal probability (khaki-left: *n* = 25; khaki-right: *n* = 25; white-left: *n* = 25; white-right: *n* = 25; see Table 2, Figure 4). Each original RSK was replicated four times for each task, resulting in a total of 800 trials (400 for the color task, 400 for the motion task).

**Table 2:** Parametric conditions used in Experiment 2.

**Exp**.	**Task**	**Motion coherence (%)**	**Color coherence (%)**
2	Motion	5, 15, 25, 35, 45, 55, 65, 75, 85, 95	55_K_, 65_K_, 75_K_, 85_K_, 95_K_, 55_W_, 65_W_, 75_W_, 85_W_, 95_W_
	Color	5, 15, 25, 35, 45, 55, 65, 75, 85, 95	55_K_, 65_K_, 75_K_, 85_K_, 95_K_, 55_W_, 65_W_, 75_W_, 85_W_, 95_W_

The letters in subscripts that follow color-coherence labels indicate the majority RSK uniform color (_K_ for khaki, _W_ for white).

As in Experiment 1, each trial began with a fixation cross (1,000 ms) followed by RSK presentation (until response or 2,000 ms). Trials were presented in two series of single-task blocks (one per task, counterbalanced across participants). Each series of single-task blocks was preceded by 16 practice trials. Each block consisted of 50 trials, the order of which was pseudorandomized with a maximum of four consecutive trials requiring the same target response button.

In the color task, the direction of soldier motion was task-irrelevant and could be congruent (motion in the same direction as the color response) or incongruent (motion in the direction opposite to the color response) with the color response. Distractor congruence was linearly spaced between 95% incongruence and 95% congruence with 10% intervals, making 20 levels signed relative to target response (negative and positive values for incongruent and congruent conditions, respectively). In the motion task, the uniform color was task-irrelevant and could be congruent (majority color indicating the same response as the coherent motion) or incongruent (majority color indicating the response opposite to the coherent motion) with the motion direction. Distractor congruence was linearly spaced between 55% and 95% incongruence and congruence with 10% intervals, making 10 levels signed relative to target response (negative and positive values for incongruent and congruent conditions, respectively).

#### Statistical analysis

Target coherence and distractor congruence were treated as continuous independent variables in multiple linear regression models with RT or accuracy as the dependent outcome. The effects of target coherence and distractor congruence were analyzed separately for motion and color tasks. In the motion task, the target coherence predictor included 10 levels (ranging from 5% to 95%) and the distractor congruence predictor included 10 levels. In the color task, the target coherence predictor included five coherence levels (ranging from 55% to 95%) and the distractor congruence predictor included 20 levels.

### Results

**Figure 5:**
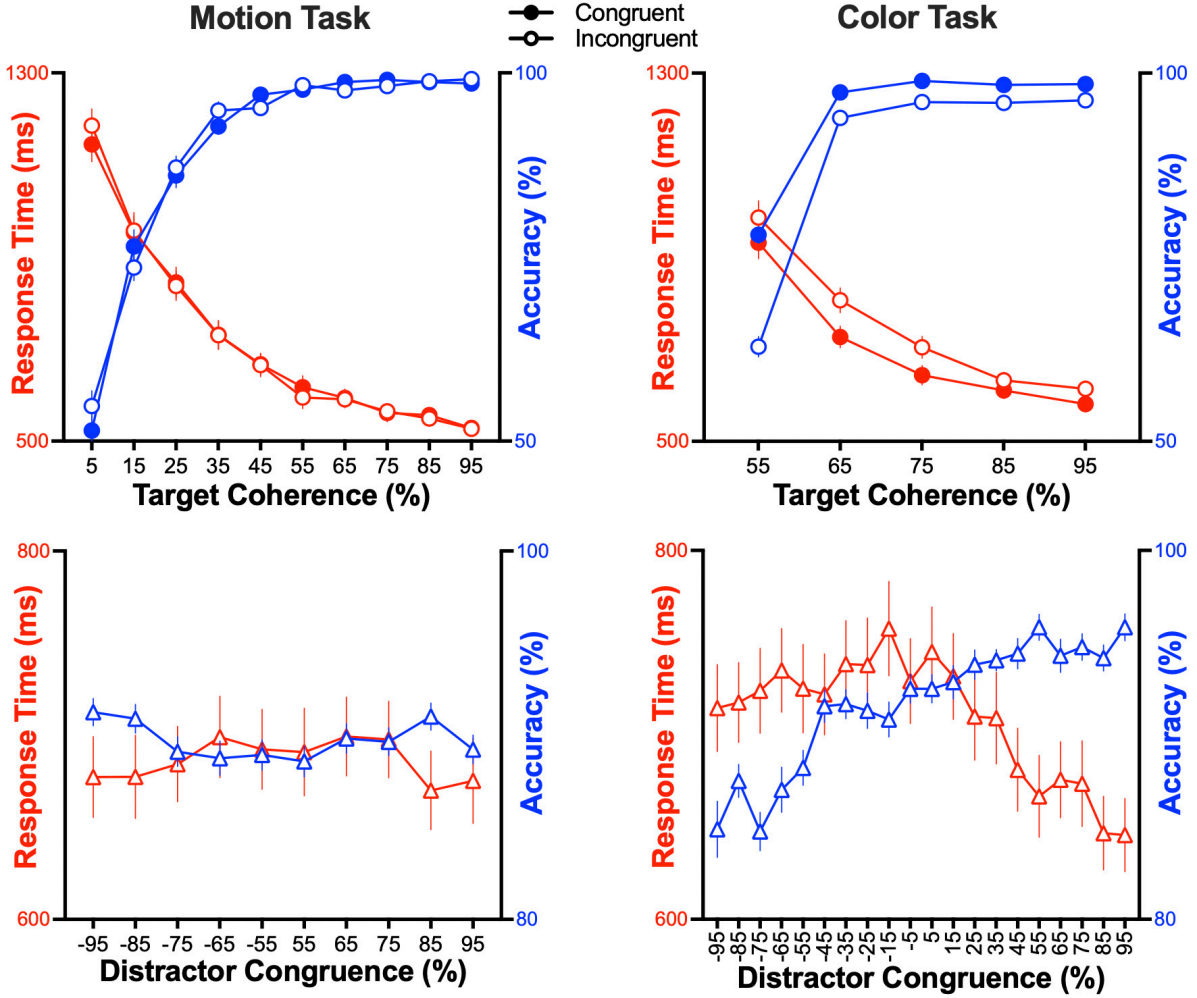
Target and distractor sensitivity to RSK stimuli. RT (red line, left axis) and accuracy (blue line, right axis) are presented as functions of target coherence **(Top)** and distractor congruence **(Bottom)**, for the motion **(Left)** and color **(Right)** tasks. In the top panels, open symbols denote incongruent stimuli, whereas filled symbols denote congruent stimuli. The percentage of soldiers moving coherently (target coherence, in the motion task) is pooled for leftward and rightward motion. The percentage of majority uniform color (target coherence, in the color task) is pooled for khaki and white uniforms. Error bars indicate the standard error of the mean.

Figure 5 depicts RT and accuracy as a function of target coherence and distractor congruence for each task. The significance of the overall regression models and regression coefficients are reported in Table 3.

**Table 3:** Regression models and regression coefficients for log-transformed RT and arc-sine transformed accuracy as dependent variables and target coherence and distractor congruence as predictors, for each task.

**Motion task**	**Predictor**	***t***	**β**	***p***	***F***	***df***	***p***	**adj. *R*^2^**
RT (log)	Target coherence	−59.68	−0.68	< 0.001				
	Distractor congruence	0.56	0.01	0.57				
	Overall model				1,781	24,196	< 0.001	0.46
Accuracy (%)	Target coherence	45.49	0.57	< 0.001				
	Distractor congruence	−0.73	−0.01	0.46				
	Overall model				1,035	24,191	< 0.001	0.33
**Color task**	**Predictor**	***t***	β	***p***	***F***	***df***	***p***	**adj**. *R*^2^
RT (log)	Target coherence	−43.3	−0.55	< 0.001				
	Distractor congruence	−9.18	−0.11	< 0.001				
	Overall model				981	24,197	< 0.001	0.32
Accuracy (%)	Target coherence	34.0	0.46	< 0.001				
	Distractor congruence	13.4	0.18	< 0.001				
	Overall model				669	24,197	< 0.001	0.24

### Motion task

Overall regression model was found to be significant for the motion task (RT: *F*_2, 4, 196_ = 1,781, *p* < 0.001, adj. *R*^2^ = 0.46; accuracy: *F*_2, 4, 191_ = 1,035, *p* < 0.001, adj. *R*^2^ = 0.33). Target coherence significantly and negatively predicted RT (β = −0.68, *p* < 0.001) and significantly and positively predicted accuracy (β = 0.57, *p* < 0.001). Distractor congruence did not significantly relate to performance (RT: β = 0.01, *p* = 0.57; accuracy: β = −0.01, *p* = 0.46).

### Color task

Overall regression model was found to be significant for the color task (RT: *F*_2, 4, 197_ = 981, *p* < 0.001; adj. *R*^2^ = 0.32; accuracy: *F*_2, 4, 197_ = 669, *p* < 0.001; adj. *R*^2^ = 0.24). Target coherence significantly and negatively predicted RT (β = −0.55, *p* < 0.001) and significantly and positively predicted accuracy (β = 0.46, *p* < 0.001). In contrast to the motion task, distractor congruence also significantly predicted changes in performance, with negative relation for RT (β = −0.11, *p* < 0.001) and positive relation for accuracy (β = 0.18, *p* < 0.001).

### Discussion

In Experiment 2, participants performed RSK tasks with stimuli involving perceptually distinct target and distractor features in single-task blocks. Target coherence and distractor congruence were independently manipulated, allowing an examination of participants' sensitivity to target and distractor information in each task.

### Target sensitivity

Regarding sensitivity to target information, we found as expected that performance was faster and more accurate with increasing levels of target coherence in both motion and color tasks. This was consistent with past decision-making research using RDK (Britten et al., 1992; Mante et al., 2013) and indicates that results obtained with RSK stimuli are similar to those obtained using classic RDK stimuli. Indeed, previous RDK studies using two-alternative forced-choice tasks reported a non-linear increase in accuracy with increasing target coherence, alongside decreases in RT that saturated at chance performance (Strittmatter et al., 2023; Spitzer et al., 2022, 2019; see also Baker et al., 1991; Lankheet and Verstraten, 1995). Our findings align with this pattern of results. To go further, we tested the difference between response accuracy and chance level at minimal target coherence for each task (using one-tailed one-sample *t*-tests). In the motion task, accuracy at 5% target coherence was at chance level for congruent trials (51 ± 13%, *t*_41_ = 0.71, *p* = 0.24, *d* = 0.11), but did not reach chance level for incongruent trials (55 ± 13%, *t*_41_ = 2.31, *p* = 0.01, *d* = 0.36). This indicates that target motion was neither detectable nor discriminable at 5% coherence with congruent conditions during the 2-s RSK lifetime. In the color task, accuracy differed from chance level at 55% target coherence for either congruent (78 ± 7%, *t*_41_ = 24.77, *p* < 0.001, *d* = 3.82) or incongruent (63 ± 9%, *t*_41_ = 9.37, *p* < 0.001, *d* = 1.45) conditions, suggesting that majority uniform color was detectable across all coherence levels. Taken together, these results indicate that the range of target coherence for the motion task (despite the above-chance incongruent condition) was sufficiently wide to allow for a comprehensive mapping of the behavioral metrics, whereas it would have been necessary to include additional target-coherence levels (below 55%) to reach chance level in the color task.

### Distractor sensitivity

Regarding sensitivity to distractor information, performance did not significantly vary with distractor congruence in the motion task. In the color task, however, distractor congruence significantly predicted changes in performance, both for RT and accuracy. In fact, participants were the fastest and most accurate when distracting motion was maximally congruent (+95% congruence: RT = 646 ± 128 ms, accuracy = 96 ± 5%), and they were slower and least accurate when distracting motion was maximally incongruent (−95% congruence: RT = 715 ± 152 ms, accuracy = 84 ± 10%), with RT and accuracy varying in a graded fashion across this continuum of interference. This demonstrated that color and motion signals influenced performance in different ways. Color coherence affected response selection solely through goal-directed decision-making (i.e., determining the correct response in the color task). In contrast, motion coherence not only affected goal-directed decision-making (i.e., determining the correct response in the motion task), but it also automatically influenced response selection when task-irrelevant, facilitating responses consistent with the direction of motion. Collectively, the results provide evidence of conflict between the tasks, necessitating selective attention for good performance (Kramer and Madden, 2008). For another perspective on task conflict, we also visualized the congruency effect relative to performance as a function of target coherence (Figure 5, open and filled symbols). This highlighted slower and more error-prone performance on incongruent than on congruent trials in the color task but not in the motion task. This difference in performance can be taken as a measure of the interference caused by soldier motion on the processing of color uniform.

### Dominance pattern of RSK tasks

The preceding results showing distractor-related response activation in the color but not in the motion task provide evidence for the dominance pattern of RSK tasks. Importantly, these findings were obtained over a series of single-task blocks where distractor information was constantly irrelevant for task-appropriate behavior and was easier to suppress in a sustained manner. Thus, color and motion tasks clearly differ in their demands for cognitive control, with the motion task being less control-demanding (dominant) and the color task more control-demanding (weaker), as it requires higher levels of control to overcome interference from conflicting motion. This affords the possibility of using RSK tasks in a Stroop-like manner (the classic case of unequal strength) in a task-switching context, where the need to switch tasks produces asymmetric switch costs resulting from the differential engagement of top-down control across the two tasks. Experiment 3 investigated this issue.

## Experiment 3

In Experiment 3, we investigated the costs of switching between RSK tasks. To this end, we employed a cued task-switching paradigm where participants were cued prior to RSK onset to apply one of two possible discrimination rules. The paradigm incorporated single- and mixed-task blocks, enabling us to examine transient and sustained components of cognitive control involved in task switching. In mixed-task blocks, participants must engage transient, trial-based control processes to reconfigure the task-set when a new task is required. Transient control processes are reflected in the performance difference between switch and repeat trials within mixed-task blocks (i.e., switch cost; Braver et al., 2003; Schwarze et al., 2024). The magnitude of the switch cost is commonly treated as an inverse measure of cognitive flexibility, with smaller switch costs indicating greater flexibility (Braem and Egner, 2018; Dreisbach and Fröber, 2019). Performing mixed-task blocks also increases the demands on cognitive control in a sustained manner, in comparison to single-task blocks, because of the need to maintain multiple task-sets in working memory. Sustained control processes are reflected in the performance difference between repeat trials in mixed-task blocks and trials in single-task blocks (i.e., mixing costs; Braver et al., 2003; Schwarze et al., 2024). Furthermore, the paradigm allowed us to examine preparatory control processes by manipulating the cue–stimulus interval (CSI; the time between task cue and RSK onset). Longer CSIs are typically associated with reduced switch costs, indicating the recruitment of preparatory (proactive) control to prepare to switch tasks in anticipation of upcoming stimuli (Dreisbach and Haider, 2006; Monsell and Mizon, 2006). Even with longer preparation time, however, a “residual switch cost” may remain due to the need for target-driven (reactive) control to resolve interference and enable successful responding (Monsell, 2003).

Employing this task-switching paradigm, the present experiment explored the costs entailed by switching between RSK tasks that are more perceptual in nature (discriminating motion or color perceptual features) and that have higher visual processing demands than the tasks classically used in task-switching studies, which are more cognitive in nature (e.g., odd/even, high/low; requiring access and, to some extent, manipulation of internal representations). Three hypotheses were formulated. First, switching between perceptual RSK features was expected to produce a transient performance cost, such that switch trials would yield slower and more error-prone responses than repeat trials (i.e., switch cost). Second, repeat trials in mixed-task blocks were expected to be slower and less accurate than trials in single-task blocks, reflecting the sustained demands of maintaining multiple task-sets (i.e., mixing cost). Both switch and mixing costs were predicted to decrease with longer CSI, as more preparation time should facilitate task-set reconfiguration. It was further expected that longer CSI would reduce but not eliminate switch costs (i.e., residual costs), since reactive control would be needed to resolve cross-task interference after RSK onset. Last, it was hypothesized that the demonstrated unequal strength between RSK tasks would cause asymmetric switch costs, as the weaker color task would require a more strongly imposed task-set, resulting in greater task-set inertia on the next trial. Consistent with the task-set inertia account (Allport et al., 1994), it was expected that switching to the motion (dominant) task would result in a larger cost than switching to the color (weaker) task.

### Method

#### Stimuli and procedure

**Figure 6:**
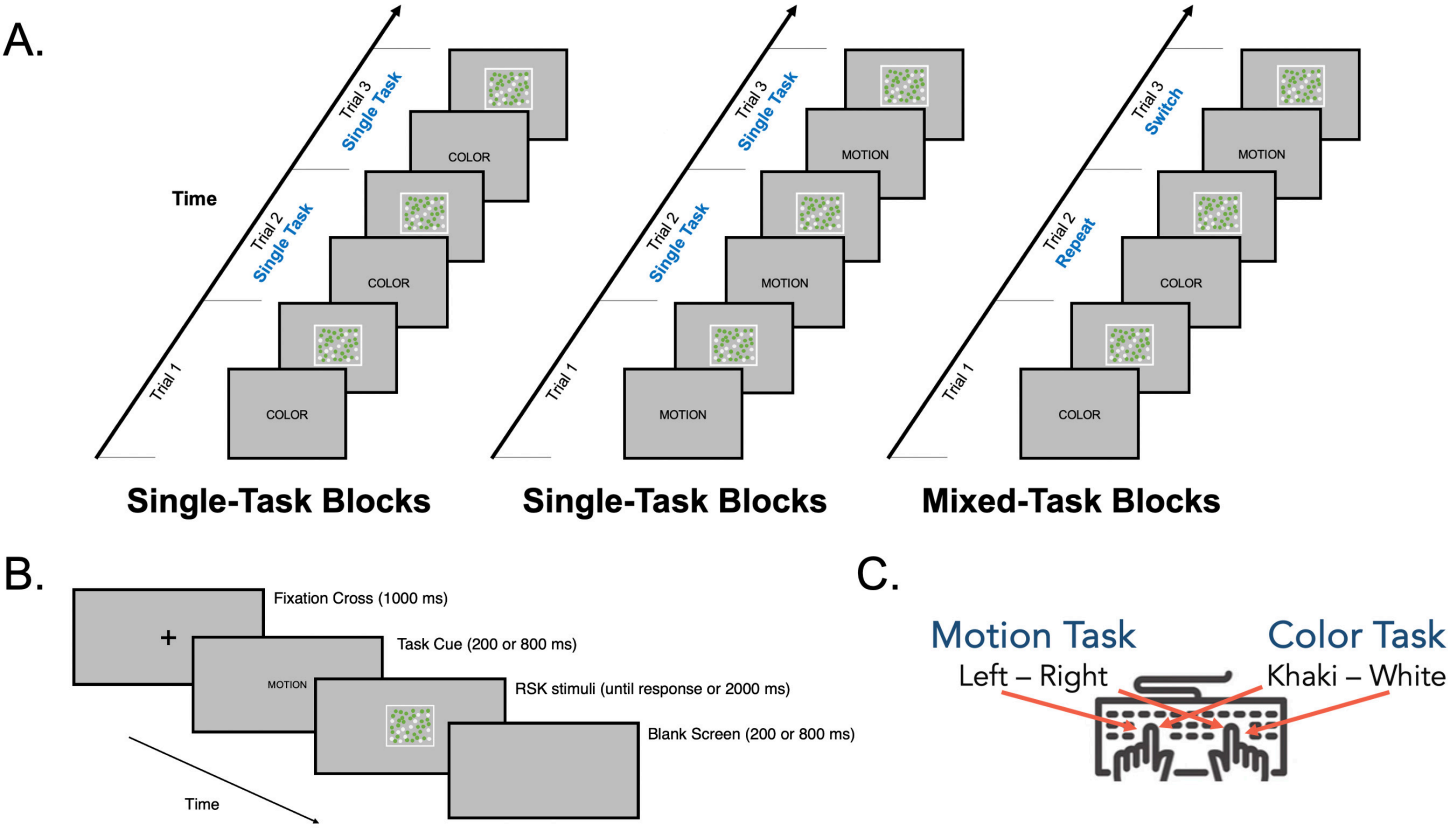
Schematic illustration of the task-switching paradigm. **(A)** In single-task blocks, the participants completed trials of the same task (i.e., single-task trials). In mixed-task blocks, motion and color tasks were intermixed, requiring participants to switch between tasks (i.e., switch trials) or to repeat the same task (i.e., repeat trials) from one trial to the next. **(B)** Procedure for trial structure. A trial started with a fixation cross lasting for 1,000 ms, followed by a cue that appeared 200 ms (short CSI) or 800 ms (long CSI) prior to RSK presentation, which lasted until the participant's response or 2,000 ms. A trial ended after a blank screen appeared, which varied in duration inversely to the CSI in order to keep the response-stimulus interval (RSI) constant between CSIs. **(C)** Stimulus-response mappings with task rules assigned to the two response hands.

RSK was systematically presented at 75% motion coherence and 75% color coherence (see Figure 3), a percentage that was considered to be easily discriminable in both dimensions based on the results of Experiment 2. A cue was presented centrally in the form of an instructional word to either discriminate the prevalent motion (French word for MOTION) or the prevalent color (French word for COLOR). Each trial consisted of (i) a fixation cross presented for 1,000 ms, (ii) task cue presentation for either 200 or 800 ms, which constituted the CSIs, (iii) RSK presentation for 2,000 ms or until response, and (iv) the presentation of a blank screen for 200 or 800 ms, depending on the CSI (Figure 6). The two CSIs were chosen to afford short (200 ms) and long (800 ms) preparation times. Importantly, the interval from the n-1 response to the n^th^ RSK was fixed at 2,000 ms (response-stimulus interval, RSI), regardless of the CSI (to control for the decay of task-set inertia; Meiran, 1996). CSI was constant within blocks but varied between blocks.

Participants performed single- and mixed-task blocks of 64 trials each. RSK majority color and coherent motion direction were equally probable in each block. In the single-task blocks, trial order was pseudorandomized with a maximum of four consecutive trials requiring the same target response button. In the mixed-task blocks, trial order was pseudorandomized with the following constraints: (i) half the trials were task-switch trials and half were task-repeat trials, (ii) switch and repeat trials were equally distributed across tasks, and (iii) the maximum was four consecutive trials of the same type (repeat, switch) or requiring the same target response button.

Tasks were administered using the “sandwich design” (Rubin and Meiran, 2005), consisting of two single-task blocks, followed by eight mixed-task blocks, and concluding with two single-task blocks (see Figure 2). First, participants completed a single-task block (either motion or color, counterbalanced across participants), preceded by 16 practice trials. Second, participants completed a single-task block of the second task, also preceded by 16 practice trials. Third, participants completed eight mixed-task blocks, preceded by 32 practice trials. Finally, participants completed two single-task blocks, identical to the second and first steps (in tasks but not in CSI), respectively. This design allowed participants to gain practice with each task before engaging in the mixed-task blocks, with single-task performance including both initial and later responses. This avoided the exclusive influence of order effects on performance. In addition, it ensured control over confounding practice effects by creating an equal number of single-task trials (256 trials: 128 color and 128 motion), repeat trials (256 trials), and switch trials (256 trials).

#### Statistical analysis

RT and error rate (rather than accuracy) are reported to align with the convention of reporting errors in task-switching studies. All participants had an error rate below 20% in each condition, so no participants were excluded based on overall performance.

The possible effects of preparation time, task sequence, and congruency on RT and error rate were assessed using 2 (CSI: short, long) × 3 (Task Sequence: single task, repeat, switch) × 2 (Congruency: congruent, incongruent) repeated-measures ANOVAs. To assess whether short CSIs resulted in significantly larger switch costs than long CSIs, a 2 (CSI: short, long) × 2 (Congruency: congruent, incongruent) repeated-measures ANOVA was then conducted on the switch-cost and error-cost dependent variables. These variables were calculated by subtracting switch trials minus repeat trials, for both RT (hereafter switch cost) and error rate (hereafter error cost) measures. The mixing-cost dependent variable (calculated by subtracting repeat trials minus single-task trials) was also entered into the same 2 × 2 ANOVA, for both RT (hereafter RT-mixing cost) and error rate (hereafter ER-mixing cost) measures. Last, possible switch-cost asymmetry was examined using a previous congruency factor (Congruency_N − 1_) indicating whether the previous trial required a more strongly imposed task-set due to conflicting responses. A repeated-measures ANOVA with a 2 (CSI: short, long) × 2 (Switch Direction: to motion, to color) × 2 (Congruency_N − 1_: congruent_n − 1_, incongruent_n − 1_) factorial design was run on the switch cost and error cost.

### Results

**Figure 7:**
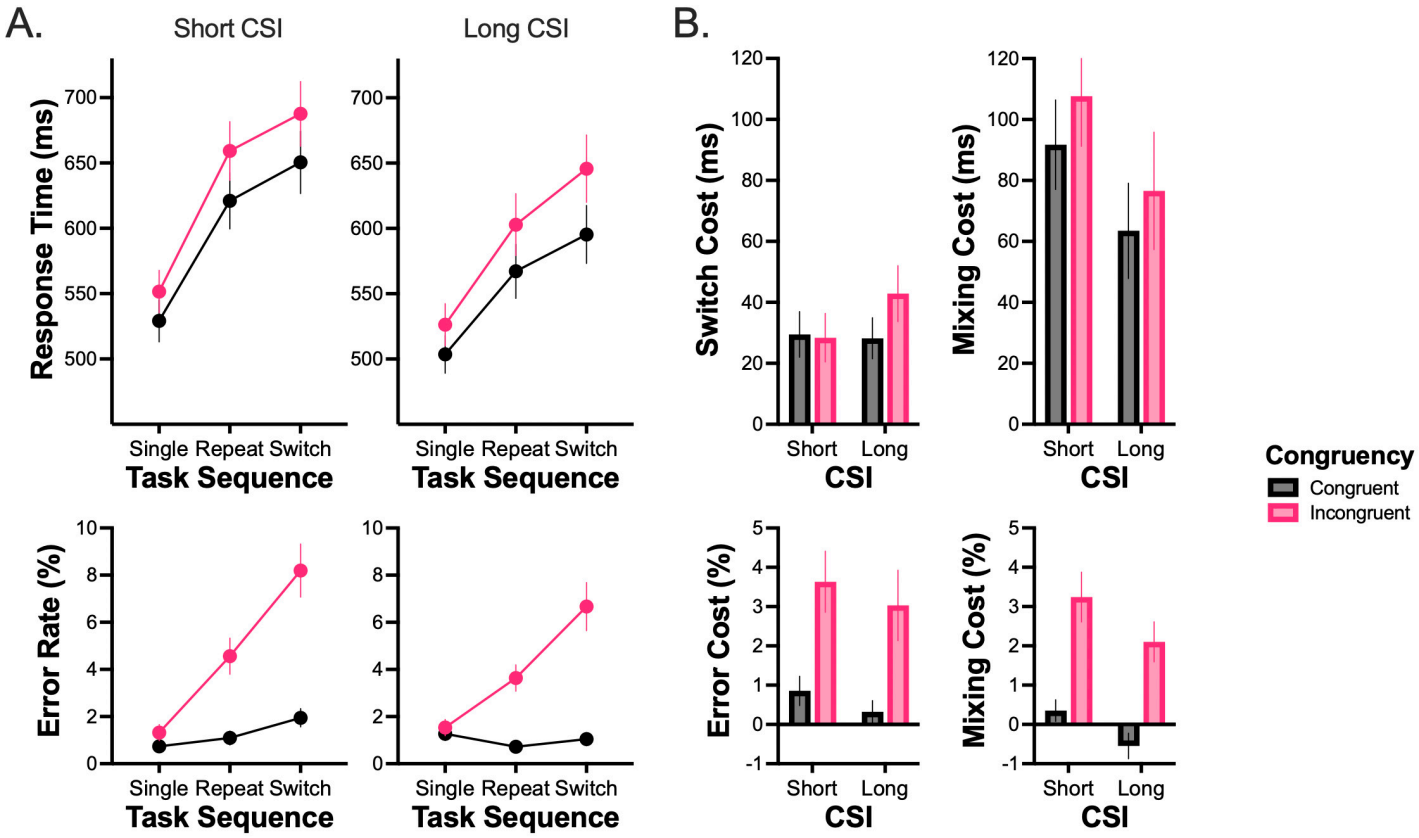
Effects of task sequence, preparation time and congruency on performance. **(A)** RT and error rate for congruent and incongruent trials as a function of task sequence and preparation time (short CSI, long CSI). **(B)** Switch cost, error cost, and mixing costs as a function of CSI and congruency. Error bars indicate the standard error of the mean.

Figure 7A depicts RT and error rate as a function of task sequence, CSI and congruency. Figure 7B depicts the corresponding switch cost, error cost, and mixing costs. Table 4 summarizes the ANOVA results establishing the cognitive costs.

**Table 4:** Summary of costs induced by task sequence and their preparatory reduction (i.e., reduction in a cost due to increased preparation time).

**Manipulation**	**Switch cost**	**Error cost**	**RT mixing cost**	**ER mixing cost**
Short CSI	< 0.05	< 0.05	< 0.05	< 0.05
Long CSI	< 0.05	< 0.05	< 0.05	ns
Preparatory reduction	ns	ns	< 0.05	< 0.05

### RT

The 2 (CSI) × 3 (Task Sequence) × 2 (Congruency) ANOVA on RT revealed significant main effects of CSI (*F*_1, 29_ = 54.18, *p* < 0.001, $\eta_{\text{p}}^{2}$ = 0.65), task sequence (*F*_1.13, 32.71_ = 51.69, *p* < 0.001, $\eta_{\text{p}}^{2}$ = 0.64), and congruency (*F*_1, 29_ = 88.37, *p* < 0.001, $\eta_{\text{p}}^{2}$ = 0.75). The interactions between CSI and task sequence (*F*_1.31, 37.88_ = 5.19, *p* = 0.020, $\eta_{\text{p}}^{2}$ = 0.15) and between task sequence and congruency (*F*_2, 58_ = 4.09, *p* = 0.022, $\eta_{\text{p}}^{2}$ = 0.12) were also significant. No other interaction effect was observed.

The CSI × Task Sequence interaction revealed that RTs for the three task sequences all differed significantly in both short CSI (single-task = 540 ± 88 ms, repeat = 640 ± 119 ms, switch = 669 ± 125 ms) and long CSI (single-task = 515 ± 83 ms, repeat = 585 ± 119 ms, switch = 621 ± 130 ms), indicating significant switch cost and RT-mixing cost. This interaction was driven by a differential effect of CSI as a function of task sequence. RT was significantly longer in the short CSI than in the long CSI on repeat trials and on switch trials, but not on single-task trials. The 2 (CSI) × 2 (Congruency) ANOVA conducted on the switch cost revealed no main or interaction effect, indicating no significant preparatory reduction in switch cost, as shown in Figure 6B. For the RT-mixing cost, the same ANOVA revealed only the main effects of congruency (*F*_1, 29_ = 6.11, *p* = 0.020, $\eta_{\text{p}}^{2}$ = 0.17) and CSI (*F*_1, 29_ = 8.18, *p* = 0.008, $\eta_{\text{p}}^{2}$ = 0.22). The main effect of CSI indicated that short CSIs produced significantly larger RT-mixing cost (100 ± 84 ms) than long CSIs (70 ± 96 ms). Regarding the Task Sequence × Congruency interaction, all *post-hoc* comparisons yielded significant differences except between repeat-incongruent trials and switch-congruent trials.

### Error rate

The ANOVA on error rate revealed significant main effects of task sequence (*F*_1.28, 37.06_ = 36.04, *p* < 0.001, $\eta_{\text{p}}^{2}$ = 0.55), congruency (*F*_1, 29 =_ 46.40, *p* < 0.001, $\eta_{\text{p}}^{2}$ = 0.62), and a significant interaction between these two factors (*F*_2, 58_ = 32.53, *p* < 0.001, $\eta_{\text{p}}^{2}$ = 0.53). The CSI × Task Sequence interaction was also significant (*F*_2, 58_ = 4.84, *p* = 0.011, $\eta_{\text{p}}^{2}$ = 0.14). No other main or interaction effects were observed.

The Task Sequence × Congruency interaction revealed that the task-sequence effects differed across congruency conditions. In the congruent condition, there were no significant error-rate differences between task sequences (single-task: 1.0 ± 1.0%; repeat: 0.9 ± 1.3%; switch: 1.5 ± 1.3%). In the incongruent condition, error rates all differed significantly (single-task: 1.4 ± 1.5%; repeat: 4.1 ± 3.1%; switch: 7.4 ± 5.4%). The CSI × Task Sequence interaction showed significantly different error rates between all task sequences in the short CSI condition (single-task: 1.0 ± 1.3%; repeat: 2.8 ± 2.6%; switch: 5.1 ± 3.8%), and between repeat (2.2 ± 1.9%) and switch trials (3.9 ± 2.9%) in the long CSI condition. These outcomes indicated significant error cost with both short and long CSIs, and a significant ER-mixing cost with short CSIs. The 2 (CSI) × 2 (Congruency) ANOVA on the error cost revealed only a significant main effect of congruency (*F*_1, 29_ = 15.34, *p* = < 0.001, $\eta_{\text{p}}^{2}$ = 0.35), thus indicating no significant preparatory reduction in error cost (see Figure 7B). For ER-mixing cost, the same ANOVA revealed only main effects of congruency (*F*_1, 29_ = 27.39, *p* = < 0.001, $\eta_{\text{p}}^{2}$ = 0.49) and CSI (*F*_1, 29_ = 4.96, *p* = 0.034, $\eta_{\text{p}}^{2}$ = 0.15). The main effect of CSI indicated that short CSIs produced significantly larger ER-mixing cost (1.8 ± 3.0%) than long CSIs (0.7 ± 2.7%).

### Switch cost and error cost asymmetry

**Figure 8:**
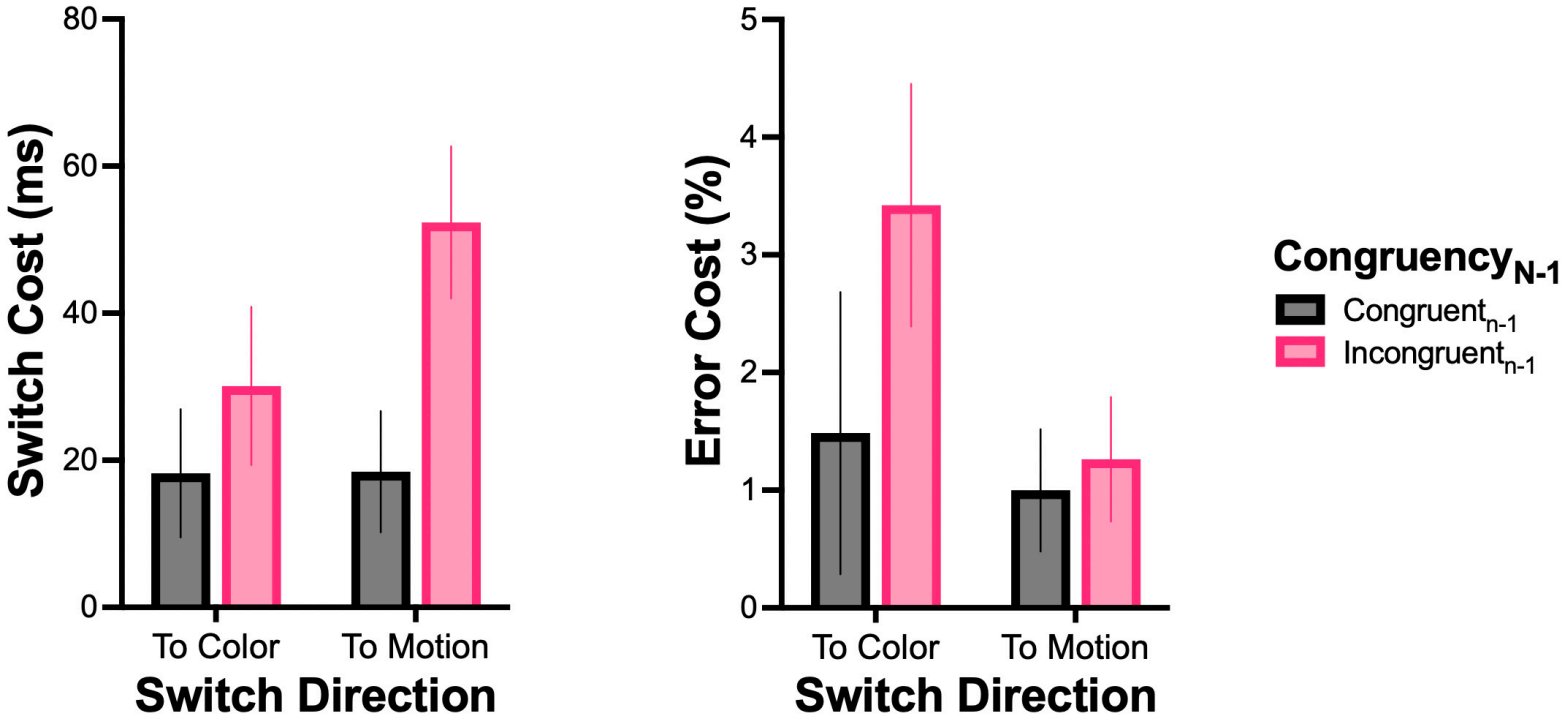
Asymmetry of switch cost and error cost. Switch cost **(Left)** and error cost **(Right)** as a function of switch direction (to color, to motion) and congruency in the previous trial (congruency_N − 1_). Error bars indicate the standard error of the mean.

Figure 8 depicts switch cost and error cost as a function of switch direction. The 2 (CSI) × 2 (Switch Direction) × 2 (Congruency_N − 1_) ANOVA on switch cost revealed a main effect of congruency_N − 1_ (*F*_1, 29_ = 13.65, *p* < 0.001, $\eta_{\text{p}}^{2}$ = 0.32), and a significant interaction between switch direction and congruency_N − 1_ (*F*_1, 29_ = 6.05, *p* = 0.020, $\eta_{\text{p}}^{2}$ = 0.17). When switching to the color task, similar costs were found between incongruent_n − 1_ (30 ± 59 ms) and congruent_n − 1_ (18 ± 47 ms) trials. When switching to the motion task, switch cost was significantly larger when switching away from incongruent_n − 1_ trials (53 ± 56 ms) than from congruent_n − 1_ trials (18 ± 45 ms), and then all other conditions. No other main or interaction effect was observed.

For error cost, the ANOVA revealed a main effect of switch direction (*F*_1, 29_ = 4.81, *p* = 0.036, $\eta_{\text{p}}^{2}$ = 0.14), indicating larger error cost when switching to the color task (2.5 ± 6.2%) than to the motion task (1.1 ± 2.9%), and a main effect of congruency_N − 1_ (*F*_1, 29_ = 4.87, *p* = 0.035, $\eta_{\text{p}}^{2}$ = 0.14), indicating larger error cost when switching away from incongruent_N − 1_ (2.4 ± 4.6%) than from congruent_n − 1_ (1.2 ± 5.0%) trials. No other main or interaction effect was observed.

### Discussion

The present experiment investigated the costs of the cognitive control processes entailed by switching between RSK tasks. Transient control processes for implementing the newly relevant task-set (reflected in switch costs; Braver et al., 2003; Schwarze et al., 2024), sustained control processes for keeping multiple task-sets in working memory (reflected in mixing costs; Braver et al., 2003; Schwarze et al., 2024), and preparatory control processes (reflected through manipulating CSI; Dreisbach and Haider, 2006; Monsell and Mizon, 2006) were investigated. Overall, the analysis demonstrated the classic effects in the task-switching literature, as significant switch costs, mixing costs, and preparation benefits were evidenced. The findings are discussed below.

### Switch cost and error cost

Participants were slower and committed more errors in switch trials compared to repeat trials, for both short and long CSIs. Switching between tasks was thus accompanied by significant switch cost and error cost even with the longer time to prepare. This observation of “residual costs” highlights reactive control demands in resolving target-driven interference after RSK onset (Karayanidis et al., 2010). As anticipated, when participants were required to make a different RSK categorization from that of the previous trial, they needed to engage transient cognitive control to retrieve the task goal, shift attention between RSK perceptual features, and retrieve the relevant S-R mapping (task-set reconfiguration, Monsell, 2003) and/or resolve interference from the previously active task-set (task-set inertia, Kiesel et al., 2010; Vandierendonck et al., 2010). These processes increased response latencies and also caused errors in the case of incomplete reconfiguration and/or failure to overcome interference from the previously active task-set. It is noteworthy that the error data strictly mirrored the latency data. This ruled out any possibility of a speed–accuracy trade-off and reinforced the evidence that the increased errors in switch trials were caused by task-set reconfiguration and/or interference from the previous irrelevant task-set.

While preparation benefits were observed for RT (not for errors) in switch trials and repeat trials (i.e., RT was significantly shorter in long CSIs compared to short CSIs, see Figure 7A), no preparatory reduction occurred (i.e., there was no significant reduction in switch cost with long CSI, see Figure 7B), indicating that the preparation benefit was not greater in switch trials than in repeat trials. These findings have two implications. First, since CSI was not confounded with the remoteness from the previous trial (CSI was manipulated while keeping the RSI constant), this implies that the decrease in RT with the longer CSI was due to proactive preparation for the upcoming task rather than passive, dissipating interference from the previous trial. Second, since the preparation benefit was not greater for switch trials than for repeat trials, this provides evidence for preparation in repeat trials and contradicts the idea of a “switch-specific preparation process” (Rogers and Monsell, 1995). This is not a novel finding in the cognitive control literature (e.g., De Jong, 2000; Nieuwenhuis and Monsell, 2002; Koch and Allport, 2006; Kiesel et al., 2010), particularly when there is an equal likelihood of switch and repetition in mixed-task blocks (see Altmann, 2004; Koch, 2001; Koch and Allport, 2006, that did not observe a reduction in switch cost with short and long CSIs). Speculatively however, this may also be due partially to the visual processing demands of RSKs, which differ from the stimuli typically used in this literature, with moving objects (soldiers), perceptual-cognitive load (the number of soldiers to monitor simultaneously), and spatial uncertainty (participants cannot know exactly where target information will appear in the square, thus requiring constant prediction and updating). This may have increased the participants' overall task readiness, regardless of whether the following trial involved switching or repeating.

Regarding congruency, participants were overall slower on incongruent trials compared to congruent trials, demonstrating a significant conflict effect due to incongruency among S-R mappings (Kornblum et al., 1990). As Experiment 2 demonstrated task conflict in the absence of task switching, this was not surprising. Importantly, the increase in error rate on switch trials compared to repeat trials was found only for the incongruent condition, indicating that the error cost was exclusive to RSKs involving response conflict. This likely occurred because there was more interference in switch trials due to participants having performed the alternative task just recently, as demonstrated in prior studies (e.g., Gilbert and Shallice, 2002; Koch and Allport, 2006). This probably led to additional cognitive processing of distractor information in the newly relevant task due to distractor-related response activation, thus increasing the impact of the distractor interference, despite its irrelevance for goal-directed decision-making (Kessler, 2017). Crucially also, as no preparatory reduction in error cost was observed, this interference was not counteracted by more task preparation, highlighting that participants were highly vulnerable to intrusion from the other task when the task sequence required switching to an incongruent RSK.

### Mixing cost

Results showed that participants were significantly slower on repeat trials than single-task trials with both short and long preparation times, demonstrating a significant RT-mixing cost. This confirmed the hypothesis that mixed-task blocks require higher cognitive control demands than single-task blocks: they incurred a penalty in RT when the same task was repeated in a multitasking rather than a monotasking context due to the underlying sustained processes of maintenance, selection and monitoring of multiple task-sets. Consistent with previous findings (Los, 1996; Meiran, 1996), mixing-cost magnitude was larger than switch-cost magnitude since it captures other abilities than performing the task switch itself and implies differences in arousal, and mental effort (Meiran, 1996; Rogers and Monsell, 1995), even for RSK perceptual discrimination tasks that do not require retrieval and manipulation of internal representations. In contrast to switch cost, RT-mixing cost was significantly larger in the short than in the long CSI, as previously found by studies using similar CSIs (100 and 1000 ms; see Lawo et al., 2012; Rubin and Meiran, 2005). This indicated that the 200-ms interval was insufficient for participants to flexibly update task-sets. This preparatory reduction in RT-mixing cost reflects more complete preparation processes that serve to update task-set representation and resolve conflict in mixed-task blocks (Mayr, 2001).

Regarding error rate, participants made more errors in repeat trials than in single-task trials in the short CSI but not in the long CSI, indicating a significant ER-mixing cost exclusive to the short preparation condition. In other terms, participants repeated RSK tasks equally well in the multitasking and monotasking contexts with a long time to prepare (no increase in error rate in repeat trials in the long CSI), highlighting that the need for flexibility in mixed-task blocks impaired the efficiency (RT) but not the effectiveness (error rate) of performance. In terms of congruency, the ER-mixing cost paralleled the error-cost results: repeat trials differed in errors from single-task trials only for incongruent RSK. Again, this points to the observation that the need for flexibility in mixed-task blocks—even when focusing on task sequences that consist of repeating the same task—increased distractor interference, leading to more errors with repeat-incongruent stimuli, thus producing overall ER-mixing cost. However, in contrast to error cost, ER-mixing cost was significantly reduced with long preparation, indicating that the processes involved in updating task-sets in mixed-task blocks did not provide efficient preparation for, and resolution of, task-set conflict in the short-CSI condition.

### Asymmetry of switch cost and error cost

As predicted, results showed a marked asymmetry of switch cost, since switching to the motion task and away from incongruent trials resulted in a larger switch cost than switching to the color task, irrespective of previous congruency. As this asymmetry was caused by conflicting motion in the previous color task, it is likely due to carryover effects of the previously active task-set (task-set inertia), as has been proposed in the task-switching Stroop literature (e.g., Allport and Wylie, 1999, 2000). In fact, larger switch cost is typically found when switching from a harder, non-dominant task (such as Stroop color naming) to an easier, dominant task (Stroop word naming), compared to the other way around (e.g., Allport et al., 1994; Monsell et al., 2000; Yeung and Monsell, 2003). This effect has been attributed to the persistence of processes that were involved in the previous harder, non-dominant task, such as inhibition of the easier, dominant task (Allport et al., 1994; Yeung and Monsell, 2003; see Kiesel et al., 2010, for a review). In the context of RSK tasks, when participants performed the color task with incongruent motion, strong inhibition of motion processing was required to counter the conflict from the irrelevant easier, dominant task. In all likelihood, this inhibition persisted and disturbed the execution of the subsequent trial in which motion was the now-relevant perceptual feature, leading to larger switch costs for motion discrimination. On the other hand, there was little or no inhibition of color processing during the motion task. Thus, no persisting inhibition needed to be overcome, resulting in comparatively smaller switch costs for color discrimination.

Regarding error cost, results showed the main effects both of switch direction, indicating more errors when switching toward the color task, and of congruency_N − 1_, indicating more errors when switching away from incongruent trials. These results do not parallel switch-cost asymmetry and are difficult to interpret, particularly as the literature has reported varied results on this issue. Several studies have indeed failed to observe asymmetric error costs (Meuter and Allport, 1999; Costa and Santesteban, 2004) or have reported effects for RTs only (Mayr and Keele, 2000; Philipp et al., 2007), failing to address whether participants traded off speed against accuracy. Despite the difficult interpretability of the error-cost asymmetry pattern, one can contend that the processes underlying switch cost and error cost asymmetry may differ. Switch-cost asymmetry may better reflect the time needed to overcome task-set inertia, which begins after a task switch but before RSK onset. On the other hand, error-cost asymmetry may better reflect task-specific processing that can only take place after RSK onset and may lead to more errors in the color task after a task switch, considering that a harder, non-dominant task (color task) logically produces more errors.

## General discussion

This article introduced RSK motion and color tasks to examine task-switching performance using stimuli that better reflect the visual processing demands of dismounted military environments.

Experiment 1 examined and ruled out the possibility that perceptual differences between uniform colors affected discrimination performance. This ensured the absence of systematic confounds that could bias the processing of target and distractor information in subsequent experiments. In Experiment 2, target coherence and distractor congruence were independently manipulated to investigate participants' sensitivity to target and distractor information. Results showed that distracting motion interfered with color discrimination, whereas distracting color did not reversely interfere with motion discrimination, demonstrating an unequal strength between the motion task (dominant, less-control demanding) and the color task (weaker, more control-demanding). Experiment 3 examined the cost of switching between RSK tasks, yielding findings consistent with the task-switching literature. First, switching between tasks led to significant switch costs and error costs, which remained similar regardless of CSI. Second, task performance was poorer when repeating the same task in mixed-task blocks compared to single-task blocks, demonstrating significant RT- and ER-mixing costs. Finally, we replicated the phenomenon of asymmetric switch costs, with a larger cost when switching to the motion task, suggesting that greater cognitive effort was required to release the motion task from its backward inhibition. We now discuss the implications of these findings in relation to the task-switching literature on perceptual tasks, and explore future applications of RSK in military contexts.

### Contribution to the task-switching literature on perceptual tasks

As task switching has been extensively explored in relation to the cognitive aspects of interpreting task stimuli (such as the retrieval and manipulation of internal representations), this study contributes to the limited body of research examining the perceptual aspects of switching between visual attributes of task stimuli.

Previously, Walther and Fei-Fei (2007) examined switching between attributes of natural scenes and found a switch cost of 23–31 ms for trials requiring attentional shifts to a different visual attribute. This switch cost was significant for a preparation time of 50 ms but not for 200 ms, consistent with Wolfe et al. (2004), who found significant switch costs up to 200-ms preparation time in a visual search task. These results support the view that an interval of 200 ms is sufficient to shift attention to the cued stimulus attribute without incurring a cost, which markedly contrasts with our findings demonstrating switch costs even with an extended preparation interval of 800 ms. Conversely, Slagter et al. (2006) cued participants to discriminate the orientation (horizontal/vertical) of a rectangle at a particular location or of a particular color and found switch costs with a preparation interval of 1,500 ms. As proposed by Walther and Fei-Fei (2007), the object-file metaphor Kahneman and Treisman, (1984) may account for these contradictory results. In the object-file metaphor, shifting attention to another object requires closing the old object file and opening a new one (i.e., a temporary representation linking and integrating successive states of an object; Kahneman et al., 1992). In contrast, as proposed by Walther and Fei-Fei (2007), shifting attention to another attribute involves accessing a different entry within the same object file, which may require less time than opening a new file. From this perspective, one could argue that the switch costs observed in Slagter et al. (2006) and in the present study are primarily driven by shifting attention to a different object rather than to a different attribute within the same object.

Visual selective attention is often classified as spatial, feature-based, or object-based (for a review, see Yantis, (2000). Since RSK stimuli were randomly generated from trial to trial, the spatial location of each soldier was unpredictable. Consequently, attentional selection during RSK trials was directed toward non-spatial visual attributes, either feature-based (prioritizing the processing of non-spatial features) or object-based (prioritizing attending to an object, which can be defined as a collection of low-level features such as coherent motion and color; see Liu, 2019). While our study does not allow us to distinguish between feature-based attention and attention to objects based on their features, one can speculatively suggest that the attentional mechanisms underlying switching between motion and color discrimination in RSK stimuli involved shifting between different objects (soldiers, defined by their respective low-level features) rather than shifting between different features within the same object (RSK). It would be worthwhile for future studies to investigate the mechanisms involved in shifting attention in relation to switch costs and their resistance to preparation, to further advance our understanding of this issue.

Second, the current study contributes to the task-switching literature on perceptual tasks by highlighting the usefulness of examining the dominance pattern of tasks included in a task-switching situation. This is relevant because evidence regarding the dominance pattern of the tasks and the mechanisms applied to control cross-task interference remains scarce in task-switching research (Jost et al., 2017), due to the common practice of aggregating data across tasks. To address this, following Ritz and Shenhav (2023), we orthogonally varied target and distractor information, measuring how participants processed these two dimensions when performing novel RSK tasks in the absence of task switching. While conflict is typically demonstrated using binary distractor congruence (congruent/incongruent; e.g., Danielmeier et al., (2011), this approach—parametrizing both target coherence and distractor congruence—provides precise measures of sensitivity to target and distractor information, offering several advantages. First, clarifying the cognitive-control demands of each task avoids speculation on relative task strength when using novel tasks, leading to more accurate predictions regarding switch cost symmetry or asymmetry. Additionally, this allows researchers to set the signal-to-noise ratio of the task-relevant stimulus dimension (target coherence) at desired levels of task difficulty and to adjust relative task strength, considering that differences in the signal-to-noise ratio between tasks may influence task dominance (see Spitzer et al., 2019). Given these advantages, we advocate for the adoption of this approach in future task-switching research with perceptual discrimination tasks.

### Future RSK usage in the military

This study demonstrated that switching between RSK tasks requires selecting target information while minimizing interference from distractor information, pitting more control-demanding responses (in the color task) against more automatic responses (in the motion task). This aligns with the cognitive demands placed on dismounted soldiers, who must constantly reconfigure task sets to switch between different tasks that may vary in their level of difficulty or automatization, depending on situational demands.

Recently, Bale et al. (2025) stated that the environments in which tactical populations operate are typically ambiguous, and that both research and training in combat shooting often fail to replicate environments with similar informational properties. They also underscored the need for future research and training practices to better sample the informational constraints and ambiguities of combat settings Bale et al., (2025). RSK could be valuable in this regard. First, it enables a flexible environment with tasks of varying difficulty levels (by varying target coherence) and interference levels (by varying distractor congruence). This expands research and training possibilities by better sampling the informational constraints surrounding soldiers, with adjustable signal-to-noise ratios of both task-relevant and task-irrelevant information. Second, it allows for the analysis of errors caused by salient (high-coherence) incongruent distractors, which present a different challenge from the difficulty posed by low-coherence targets that make correct responses harder to determine. Third, it can be integrated into shooting simulators involving actual shooting responses toward colored targets positioned on either side of the RSK (e.g., a khaki-colored target on the left and a white-colored target on the right). This may be useful for exposing soldiers to the complexities of dismounted environments, where shooting decisions in sequential tasks can rely on noisy information and/or occur under interference.

It warrants mention, however, that the potential of the RSK as a training tool has yet to be established. This will require future experiments that compare RSK-based task-switching training with alternative training approaches and demonstrate transfer to real-world dismounted environments. Hence, while this study demonstrates that the RSK as a reliable laboratory tool for studying task switching under controlled informational constraints on visual processing, we emphasize that performance in motion-color RSK tasks should not be considered a proxy or a predictor of operational sequential multitasking performance.

### Limitations

The present findings should be interpreted in light of several limitations. First, RSK tasks involved minimal motor demands (key press) and did not reflect the full-body responses required of soldiers. As a result, response selection and execution processes were serial: task conflict had to be resolved before the response was executed (Quétard et al., 2023). However, response selection in ecological settings can continue to evolve during movement execution (i.e., after movement response initiation; see Quétard et al., 2023). This overlap between selection and execution processes may contribute to soldiers' performance in dismounted environments, limiting the generalizability of our results to applied military contexts.

Second, the present study did not account for position in run (i.e., the ordinal position of a trial within a sequence of task repetitions) and treated all task-repeat trials as equivalent. Since performance tends to improve gradually across successive repetitions of the same task (i.e., repetition benefits; see Bonnin et al., 2011), the reported effects may conflate switch-related processes with repetition-related improvements. Future work modeling this factor could help distinguish switch-specific costs entailed by shifting attention between RSK perceptual features from performance changes across repetitions.

Third, while RSK tasks with lower coherence levels were intrinsically more difficult, they also likely required greater cognitive effort (i.e., the depth of engagement with a cognitive task; see Braver, 2012; Shenhav et al., 2013). Although Experiment 3 was conducted with fixed coherence parameters (thereby eliminating potential confounds due to effort–difficulty covariation), this raises the question of whether switching between RSK tasks with lower coherence levels (probably more effortful) would yield similar switch costs in terms of magnitude and resistance to preparation. Future experiments should aim to disentangle participants' cognitive effort from RSK task difficulty to clarify this issue.

Finally, it would be useful to investigate the neural mechanisms underlying switching between RSK tasks. This would enable linking asymmetric switch costs to neurophysiological markers reflecting differential temporal dynamics of cognitive control as a function of switch direction (for an example with letter stimuli, see Wu et al., 2015). Such insights would advance our understanding of the demands imposed by the RSK task-switching paradigm and inform considerations of its potential utility for future applications in the military.

## Data Availability

The raw data supporting the conclusions of this article will be made available by the authors, without undue reservation.

## Footnotes

1 In multitasking research, a basic distinction concerns whether the situation requires performing two tasks concurrently (i.e., dual-tasking) or sequentially (i.e., task switching; see Salvucci and Taatgen, 2010). Concurrent multitasking involves performing two tasks simultaneously and yields “dual-task costs”. Sequential multitasking involves performing different tasks one after another and yields “switch costs”. In the present article, sequential multitasking refers to situations in which two tasks are performed sequentially and require switching back and forth between tasks, as conventionally examined in task-switching paradigms.

## Supplementary material

Video 1 Exp1a_MotionTask_Khaki.

Video 2 Exp1a_MotionTask_White.

Video 3 Exp1b_ColorTask.

Video 4 Exp2_MotionTask_ColorTask.

Video 5 Exp3_TaskSwitching_LongCSI.

Video 6 Exp3_TaskSwitching_ShortCSI.
